# Data-driven prediction of the shear capacity of ETS-FRP-strengthened beams in the hybrid 2PKT–ML approach

**DOI:** 10.1038/s41598-023-47064-1

**Published:** 2023-11-14

**Authors:** Thai Son Tran, Boonchai Stitmannaithum, Linh Van Hong Bui, Thanh-Truong Nguyen

**Affiliations:** 1 Research and Development Department, IRIS Technology, 301 Daeyang AI Center, 209 Neungdong-ro, Gunja-dong, Gwangjin-gu, Seoul, 05006 South Korea; 2 Center of Excellence in Innovative Construction Materials, Department of Civil Engineering, Faculty of Engineering, Chulalongkorn University, 254 Phayathai Road, Pathumwan, Bangkok, 10330 Thailand; 3 Laboratory for Computational Civil Engineering, Institute for Computational Science and Artificial Intelligence, Van Lang University, Ho Chi Minh City, Vietnam; 4 Faculty of Civil Engineering, School of Technology, Van Lang University, Ho Chi Minh City, Vietnam; 5 Industrial Maintenance Training Center, Ho Chi Minh City University of Technology (HCMUT), Ward 14, District 10, Ho Chi Minh City, Vietnam; 6 Vietnam National University Ho Chi Minh City, Linh Trung Ward, Thu Duc City, Ho Chi Minh City, Vietnam

**Keywords:** Civil engineering, Computational science

## Abstract

A new approach that combines analytical two-parameter kinematic theory (2PKT) with machine learning (ML) models for estimating the shear capacity of embedded through-section (ETS)-strengthened reinforced concrete (RC) beams is proposed. The 2PKT was first developed to validate its representativeness and confidence against the available experimental data of ETS-retrofitted RC beams. Given the deficiency of the test data, the developed 2PKT was utilized to generate a large data pool with 2643 samples. The aim was to optimize the ML algorithms, namely, the random forest, extreme gradient boosting (XGBoost), light gradient boosting machine, and artificial neural network (ANN) algorithm. The optimized ANN model exhibited the highest accuracy in predicting the total shear strength of ETS-strengthened beams and ETS shear contribution. In terms of predicting the total shear strength of ETS-strengthened beams, the ANN model achieved R^2^ values of 0.99, 0.98, and 0.96 for the training, validation, and testing data, respectively. By contrast, the ANN model could predict ETS shear contribution with high accuracy, with R^2^ values of 0.99, 0.99, and 0.97 for the training, validation, and testing data, respectively. Then, the effects of all design variables on the shear capacity of the ETS-strengthened beams were investigated using the hybrid 2PKT–ML. The obtained trends could well appraise the reasonability of the proposed approach.

## Introduction

The degradation of existing reinforced concrete (RC) structures and ways to prolong their service life require suitable intervention methods. Furthermore, the maintenance of structures requires sufficient knowledge and understanding of the behavior and performance of the structures before and after the upgrading process. This information gap leads to the unpredictable collapse of structures even though they have been strengthened by traditional systems.

Various strengthening systems built of steel-reinforced grout (SRG), steel-reinforced polymer (SRP), and fabric-reinforced cementitious matrix (FRCM) have been widely developed to rehabilitate or enhance the performance of RC structures. Examples are the works of Thermou et al.^1^, Santis et al.^2^, and Mandor and Refai^3^. In general, their studies revealed that the application of strengthening systems significantly increases both the strength and deformability of RC members. Additionally, the aforementioned retrofit methods benefit from material availability and simple procedures. However, given the thickness of retrofit elements, the beams strengthened by SRG, SRP, and FRCM materials tend to enlarge specimen geometries. Furthermore, strengthening composites may require the use of steel meshes, leading to possible corrosion and high thermal conduction.

Fiber-reinforced polymers (FRPs) have emerged as a new and effective composite in the construction industry in the last few decades. Widely known FRP materials include glass FRP (GFRP), aramid FRP, basalt FRP, and carbon FRP (CFRP). In addition, a new hybrid carbon/glass FRP was developed by Ibrahim et al.^4^ to strengthen shear-deficient beams. The prominent advantages of FRP materials compared with other materials (e.g., SRG, SRP, and FRCM) include high tensile strength, noncorrosiveness, low thermal conduction, and lightweight features. FRP materials have been widely applied in strengthening and retrofitting RC components, including beams, slabs, and columns. Moreover, FRP materials can be flexibly used as a reinforcement system for the partial/entire replacement of conventional steel in RC members^5–8^.

Among the failure modes of RC beams, shear collapse is one of the most critical and dangerous situations. Predicting the shear mechanism of RC elements remains challenging for researchers and designers. Two common shear strengthening techniques with FRP for RC beams that have been developed for certain time periods are external bonding (EB) and near-surface mounting (NSM)^9–16^. Other effective techniques have also been developed, including the use of near-surface embedded (NSE) and hybrid NSE/EB for the shear strengthening of RC beams (Wakjira and Ebead^17,18^). The aforementioned techniques require the bonding of FRP elements (laminates, strips, plates, sheets, and bars) onto the preroughed concrete surfaces or concrete grooves in the shear zone of beams by using an adhesive resin^9–16,19,20^. Although the effectiveness of retrofit methods has been studied^21–23^, the early loss of adherence of FRP to concrete continues to play an important negative role in diminishing the strengthening efficiency of the aforementioned retrofit techniques. Consequently, the embedded through-section (ETS) or deep embedment method has been experimentally investigated by researchers such as Valerio et al.^24^, Challal et al.^25^, Mofidi et al.^26^, Barros et al.^27^, Breveglieri et al.^28,29^, Bui et al.^30^, Sogut et al.^31^, and Bui et al.^32^. The ETS technique requires inserting and adhering the FRP elements into concrete holes premanufactured along the section height in the shear span of the beams. Challal et al.^25^ and Bui et al.^32^ found that the ETS-FRP strengthening system outperforms the EB-FRP and NSM-FRP retrofit systems.

Numerical research on RC beams repaired/retrofitted with EB-FRP or NSM-FRP elements has been broadly examined^33–43^. However, excluding the experimental studies, only a few analytical and numerical investigations on the shear responses of ETS-FRP-strengthened RC beams have been conducted^44–50^. Previous studies^33–50^ focused on the finite element method (FEM) and the development of design models. Although the experimental results demonstrate the accuracy and agreement of previous findings, FEM simulation entails costly computational packages and complicated models^33–50^.

A number of studies^44,46,49^ have proposed the closed-forms of shear strength models of RC beams strengthened with FRP composites by referring to common theories such as truss analogy and strut-and-tie. Wakjira and Ebead^51^ proposed a simplified compression field theory-based model to predict the shear behavior of FRCM-strengthened RC beams and found that its accuracy and reliability were higher than those of other shear models. Recently, analytical approaches for capturing the full shear load with respect to deflection/deformation of FRP-strengthened RC beams have been developed^52–54^. Among them, the two-parameter kinematic theory (2PKT) proposed by Mihaylov^55^ is a simple and powerful model for simulating the entire load‒deflection curve of RC beams. The 2PKT approach considers two degrees of freedom (DOFs), as defined by the displacement of the critical loading zone (*Δ*_*c*_) and the average strain in the bottom reinforcement (*ε*_*t,avg*_), in the formulation of model equations. The calculation speed of the 2PKT approach is fast and not complicated to use. The accuracy and suitability of the 2PKT have been demonstrated in the prediction of full shear behaviors of conventional steel-/FRP-RC beams and retrofitted RC beams^56–59^. However, no ETS-FRP-strengthened RC beams have been simulated with the 2PKT approach. Moreover, the bonding-based model proposed in Bui et al.^32^ for simulating the shear mechanism of FRP strengthening systems adhered to concrete has not been integrated into 2PKT modeling. The integration of the bonding-based model into the original 2PKT approach is expected to enhance the simulation of the full shear response of ETS-FRP-strengthened RC beams.

Recently, machine learning (ML) algorithms have increasingly been used in civil engineering to predict the behaviors of concrete materials or structures. Liang et al.^60^ employed various ML models to predict the creep behavior of concrete. Chakraborty et al.^61^ utilized an ML model to forecast the compressive behavior of high-performance concrete. Mangalathu et al.^62^ used ML to classify the failure mode and predict the shear strength of RC beam-column joints. Solhmirzaei et al.^63^ utilized ML to predict the failure mode and shear capacity of ultra high-performance concrete beams. The aforementioned works, as well as the studies of You et al.^64^, Li et al.^65^, Lee et al.^66^, Rahman et al.^67^, and Zhang et al.^68^, have shown that ML is highly effective in accurately predicting structural behaviors. Other studies have also been conducted to predict the capacity of strengthened beams. Wakjira et al.^69^ used ML methods to predict the shear capacity of RC beams strengthened with inorganic composites. Wakjira et al.^70^ developed a tool to predict the flexural capacity of FRP-RC beams based on the result of an optimized ML model. The same authors used a data-driven approach to determine the load and flexural capacities of RC beams strengthened with FRCM composites^71^. Then, the ML method was successfully applied in predicting the behaviors of concrete materials and element structures (i.e., strengthened beams). A critical feature of ML techniques is their ability to capture complex nonlinear relationships between predictors and variables; determining this relationship if challenging when traditional methods are used. Finally, the literature^72–75^ presented data-driven models to predict the structural behavior of RC columns in terms of plastic length, shear strength, and drift capacity.

The use of ML in predicting the behavior of concrete members has been generally successful, but using ML models to predict the shear behavior of FRP-strengthened beams still needs to be further studied. Moreover, a reliable database must be established, as data on the shear behavior of RC beams strengthened with ETS technology remain inadequate. This issue may be addressed by conducting FEM simulations to help produce a database for applying ML models. However, the cost and complexity of running FEM software or programs limit its popularity among users. The 2PKT method can generate an extensive database despite its simple application and short computation time. Combining 2PKT with ML to forecast the shear capacity of ETS-FRP-strengthened beams is also promising. The combination can lead to a much more robust and accurate prediction of the shear resistance of ETS-FRP-strengthened beams and their convenient use, overcoming the basic challenge of 2PKT. For example, given a set of specific circumstances and demands, the combined model can assist data scientists with expertise in ML to perform a shear resistance assessment of ETS-strengthened RC beams even if they do not have a rigid background in civil engineering.

The present study is organized in five sections. First, the 2PKT approach is developed to successfully model the shear behavior of RC beams strengthened with ETS-FRP bars. Second, the reliability of the developed 2PKT model is validated against the experimental data of ETS-strengthened RC beams reported in the literature. Third, a data pool regarding the shear strength of ETS-strengthened RC beams is generated using the developed 2PKT approach. Fourth, a variety of rigorous ML models are applied for training and testing with the simulated 2PKT data to determine the most suitable approach for ML–2PKT combination. Finally, parametric studies on the effects of all design variables for ETS-strengthened beams are implemented using the hybrid 2PKT–ML approach.

## Development of the 2PKT approach

### Original 2PKT approach

**Figure 1:**
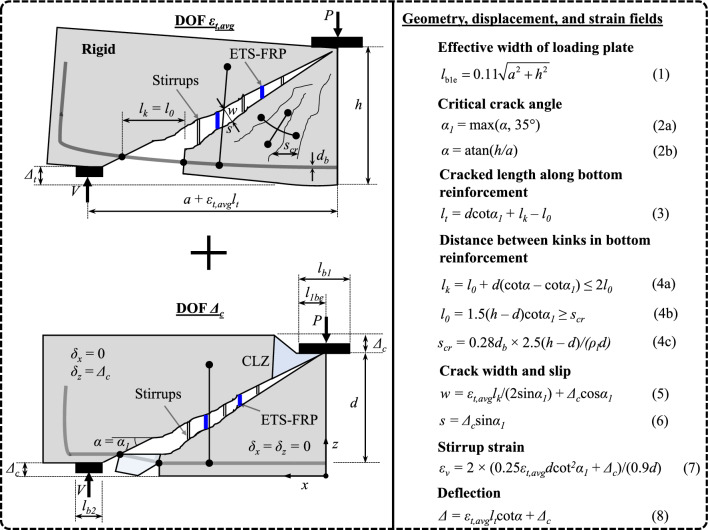
Summary of the 2PKT approach^54,57^.

The formulations of the 2PKT approach for analysis of the RC beams are described with two DOFs, which are the average strain in steel tension reinforcement (*ε*_*t,avg*_) and displacement of the critical loading zone, CLZ, (*Δ*_*c*_). Figure 1 shows the DOFs *ε*_*t,avg*_ and *Δ*_*c*_. The average strain in the longitudinal bars induces a shear critical crack that divides the shear zone of the beams into two parts: a rigid block located above the critical crack and a crack fan located below the critical crack. Meanwhile, the DOF* Δ*_*c*_ induces the vertical displacement of the beams without curvature. On the basis of the geometrical relations and kinematic characteristics, the geometries of the effective width of the loading plate (*l*_*b1e*_), crack angles (*α*, *α*_*1*_), cracked length along the bottom reinforcement (*l*_*t*_), distance between kinks in the bottom reinforcement (*l*_*k*_, *l*_*0*_), and crack spacing (*s*_*cr*_) are determined, with the formulations given by Eqs. (1)–(4c). Then, the crack width (*w*) and concrete slip at critical crack (*s*) are expressed by Eqs. (5) and (6), respectively. The strain in the steel stirrups (*ε*_*v*_) is determined using the elongation along the crack, as shown in Eq. (7). In addition, the deflection of the beams subjected to three-point bending is calculated by Eq. (8), which is composed of the superposition of the displacement caused by the curvature and deformation of the critical loading zone.

According to previous studies^57–59^, the shear strength of a conventional RC beam consists of four shear components: critical loading zone (*V*_*clz*_), aggregate interlock (*V*_*ci*_), steel stirrups (*V*_*s*_), and dowel action of the bottom reinforcement (*V*_*d*_). A number of studies^54–59^ have detailed the mechanisms of the four shear components; therefore, in this section, only the equations for deriving the shear components are summarized. The shear strength attributable to the critical loading zone (*V*_*clz*_) is written as follows:

9 $$V_{clz} = k\sigma_{avg} \left[ {\varepsilon_{max} \left( {\Delta_{c} } \right)} \right]bl_{b1e} \sin^{2} \alpha ,$$

where *k* is the crack shape factor, where *k* = min{max[1 – 2(cot*α* – 2), 0], 1} is for the deep beams (shear span-to-effective depth, *a/d* ≤ 2.5) (Fathallal et al.^54^), and *k* = min{1.5/[1 + (200*ε*_*t,avg*_cot*α*)^2^], 1} is for the slender beam (*a/d* > 2.5) with the possible presence of an S-shaped crack (Mihaylov^59^); *b* = the beam width (mm), *b* = *b*_*f*_ is the flange width with a T-shaped beam section (mm); and *σ*_*avg*_ is the average compressive stress in the CLZ (MPa), which can be derived as follows:

10 $$\sigma_{avg} = \frac{{\int\limits_{0}^{{\Delta_{c} /\left( {3l_{b1e} } \right)}} {\sigma \left( \varepsilon \right)d\varepsilon } }}{{\Delta_{c} /\left( {3l_{b1e} } \right)}},$$

where *σ*(*ε*) is the constitutive stress–strain relationship proposed by Popovics^76^.

The shear resistance caused by the aggregate interlock (*V*_*ci*_) is determined via the shear stress of the aggregate interlock (*ν*_*ci*_) as follows:

11a $$V_{ci} = 0.18\nu_{ci} (w,s)bd,$$

11b $$\nu_{ci} = 0.635\int\limits_{ - \pi /2}^{\pi /2} {\sigma_{con} K\sin \varphi \cos \varphi d\varphi } ,$$

11c $$\sigma_{con} = 0 \le 13.7\sqrt[3]{{f{^\prime}_{c}}}\frac{s \cdot \sin \varphi - w \cdot \cos \varphi }{{0.04}} \le 13.7\sqrt[3]{{f{^\prime}_{c} }},$$

11d $$K = 1{-}\exp \left( {1{-}0.5a_{g} /w} \right) \ge 0,$$

where* w* = the critical crack width (mm); *s* is the concrete slip (mm); *d* is the effective depth (mm); *φ* is the aggregate interlocking angle (radian); and *a*_*g*_ is the maximum aggregate size (mm).

The shear strength provided by the stirrups is expressed as follows:

12a $$V_{s} = \sigma_{v,avg} \rho_{v} b\left( {0.9d} \right)\cot \alpha_{1} ,$$

12b $$\sigma_{v,avg} \, = \, \left\{ \begin{gathered} E_{sw} \varepsilon_{v} /2 \, if \, \varepsilon_{v} \le \varepsilon_{yv} \, \hfill \\ \frac{{f_{yv} \varepsilon_{yv} /2 + f_{yv} \left( {\varepsilon_{v} - \varepsilon_{yv} } \right)}}{{\varepsilon_{v} }} \, if \, \varepsilon_{v} > \varepsilon_{yv} \hfill \\ \end{gathered} \right.,$$

where *E*_*sw*_ is the elastic modulus of the steel stirrups (GPa); *ε*_*yv*_ is the yield strain of the steel stirrups; and *f*_*yv*_ is the yield strength of the steel stirrups (MPa).

The shear resistance caused by the dowel action of the steel tension bars is stipulated as follows:

13 $$V_{d} = \min \left\{ {n_{b} \frac{{12E_{s} \pi d_{b}^{4} }}{{64l_{k}^{3} }}\Delta_{c} ,\;n_{b} f_{ys} \frac{{d_{b}^{3} }}{{3l_{k} }}\left[ {1 - \left( {\frac{{\varepsilon_{t,avg} }}{{\varepsilon_{ys} }}} \right)^{2} } \right]} \right\},$$

where *n*_*b*_ is the number of longitudinal tension bars; *E*_*s*_ is the elastic modulus of steel tension reinforcement (GPa); *d*_*b*_ is the bar diameter of steel tension bars (mm); *f*_*ys*_ is the yield strength of the bottom reinforcement (MPa); and *ε*_*ys*_ is the yield strain of the bottom reinforcement.

### Shear contribution of ETS-FRP strengthening system.

Regarding the EB and near-surface-mounting methods, a number of shear models to estimate the shear resisting force of FRP strengthening in the strengthened beam have been developed. For beams with ETS-FRP bars, the models attempt to predict the ETS-FRP shear contribution, but the actual measurement is underestimated^26,30^. Bui and Stitmannaithum^44^ proposed the bonding-based approach to simulate the shear resisting mechanism between ETS strengthening bars and concrete in ETS-retrofitted beams. Bui et al.^46^ and Bui and Nguyen^48^ continuously developed a new step of the bonding-based approach to analyze strengthened beams with rectangular and T-shaped sections. The corroborations to the experimental database in their studies have demonstrated the accuracy and effectiveness of the bonding-based approach for assessing the shear capacity of ETS-strengthened beams. In this section, the formulations established in the previous literature^46,48^ for the bonding-based approach are utilized for the convenience of users.

In the bonding-based approach, the shear resisting mechanism of ETS strengthening to concrete is considered by assessing their respective bond performance. A crack plane occurs in the ETS-strengthened beam, which passes through the existing steel stirrups and ETS strengthening bars, dividing the beam into two parts. The shear reinforcement restrains the shear crack opening. The anchorage hook and closed shape govern the shear resistance of the steel stirrups. Meanwhile, the contribution of the ETS strengthening system to the beam shear resistance is governed by the interfacial profile of the ETS bar-to-concrete adhered joint.

**Figure 2:**
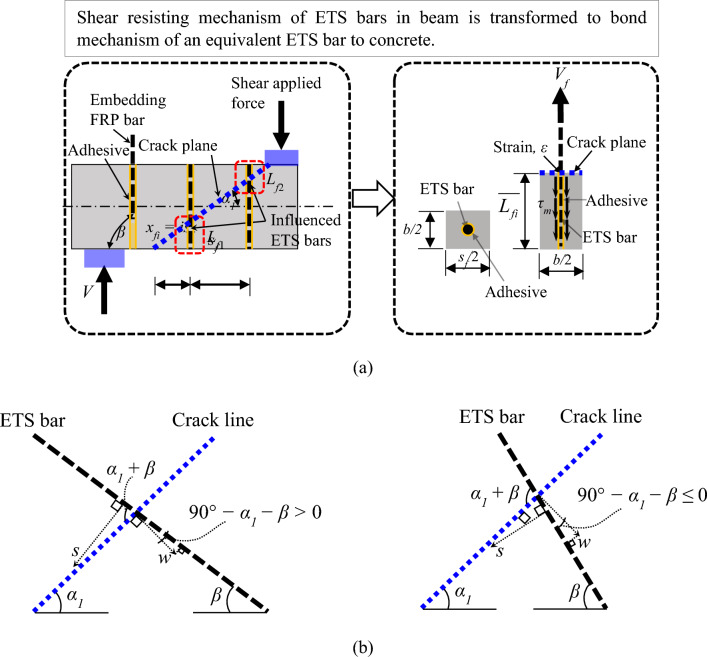
Bonding-based approach for ETS-strengthened beams: (**a**) conceptual scheme (Bui et al.^46^) and (**b**) geometrical formulation for slip between ETS bar and concrete.

Figure 2a presents the ETS technique for inserting FRP bars into the prepared holes through the beam section and bonding them with concrete by adhesive resin. The conceptual scheme of the bonding-based approach for the ETS-strengthened beams is also illustrated in the figure. The number of influenced ETS bars that are crossed by the crack line is calculated as follows:

14 $$N_{f} = {\text{round}}\;{\text{off}}\left[ {h\frac{{\cot \alpha_{1} + \cot \beta }}{{s_{f} }}} \right],$$

15 $$L_{fi} = \left\{ \begin{gathered} is_{f} \frac{{\sin \alpha_{1} }}{{\sin \left( {\alpha_{1} + \beta } \right)}}\;{\text{for}}\;x_{fi} < \frac{h}{2}\left( {\cot \alpha_{1} + \cot \beta } \right) \hfill \\ L_{f} - is_{f} \frac{{\sin \alpha_{1} }}{{\sin \left( {\alpha_{1} + \beta } \right)}}\;{\text{for}}\;x_{fi} \ge \frac{h}{2}\left( {\cot \alpha_{1} + \cot \beta } \right) \hfill \\ \end{gathered} \right.,$$

where *x*_*fi*_ = *is*_*f*_ is the distance from the end of the main crack plane to the end of the *i*^th^ single bar crossing the critical crack plane (mm),* s*_*f*_ is the ETS spacing (mm), *h* is the beam height (mm); and *α*_*1*_ and *β* represent the crack angle and the ETS system inclination (°), respectively.

The average bond length of the influenced ETS bars is given by

16 $$\overline{{L_{fi} }} = \frac{1}{{N_{f} }}\sum\limits_{i = 1}^{{N_{f} }} {L_{fi} } .$$

Bui et al.^46^ proposed a nonlinear bond model to describe the bond profile between ETS bars and concrete and developed the ETS bond model via regression and mathematical analyses. Equation (17a) represents the regression fitting of the *ε*–*s*_*ETS*_ curve from the pullout tests; Eq. (17b) represents the governing equation of the ETS bond profile; and Eq. (17c) shows the ETS bond fracture energy (*G*_*f*_). The reliability of their proposed bond law has been validated via several pullout tests of ETS bars bonded to concrete joints^46,77^.

17a $$\varepsilon = ds_{ETS} /dx = A\left[ {{1 }{-}exp\left( {{-}Bs_{ETS} } \right)} \right],$$

17b $$\tau = \left( {E_{f} A_{f} /p_{f} } \right) \times \left( {d^{{2}} s_{ETS} /dx^{{2}} } \right) = A^{{2}} B \times \left( {E_{f} A_{f} /p_{f} } \right) \times exp\left( {{-}Bs_{ETS} } \right) \times \left[ {{1}{-}exp\left( {{-}Bs_{ETS} } \right)} \right],$$

17c $$G_{f} = \int\limits_{0}^{{s_{ETS} }} {\tau ds = \frac{{E_{r} A_{r} }}{{p_{r} }}A^{2} } \left( {\frac{1}{2}e^{{ - 2Bs_{ETS} }} - e^{{ - Bs_{ETS} }} + \frac{1}{2}} \right),$$

where* ε* is the strain in the ETS bar; *A* is the bond factor representing the maximum strain in the ETS bar; *B* = ln(2)/*s*_*m*_ is the bond ductility index (1/mm);* E*_*f*_ is the elastic modulus of the ETS bar (GPa); *s*_*m*_ is the maximum slip at the peak bond stress of the ETS bar–concrete interface (mm), which simply takes the value of 0.05 mm when *E*_*f*_ > 50 GPa and 0.12 mm when *E*_*f*_ ≤ 50 GPa; *A*_*f*_ is the cross-sectional area of the ETS bar (mm^2^); *p*_*f*_ is the perimeter of the ETS bar (mm); *s*_*ETS*_ is the slip between ETS bar and concrete (mm); and *G*_*f*_ is the bond fracture energy (N/mm).

Figure 2b shows the geometrical description of the relations between the concrete slip (*s*) and crack width (*w*) at the intersection of the crack line and ETS bar. Two scenarios are considered when determining the slip of the ETS bar to concrete (*s*_*ETS*_). The dependency of the ETS bar slip on the concrete slip and crack width can be described as follows:

18a $$s_{ETS} = w{\text{cos}}\left( {{9}0^\circ {-}\alpha_{1} {-}\beta } \right){-}s{\text{cos}}\left( {\alpha_{1} + \beta } \right){\text{if}}\,\alpha_{1} + \beta < {9}0^\circ ,$$

18b $$s_{ETS} = w{\text{cos}}\left( {{9}0^\circ {-}\alpha_{1} {-}\beta } \right) + s{\text{cos}}\left( {{18}0{-}\alpha_{1} {-}\beta } \right){\text{if}}\;\alpha_{1} + \beta \ge {9}0^\circ .$$

The bonding-based approach requires information about the maximum bond stress between ETS strengthening and concrete in the strengthened beam. Bui et al.^46^ and Bui and Nguyen^48^ provided the following expressions for the maximum bond stress between ETS bars and concrete based on the average anchorage length categorization:

19 $$\tau_{m} = \left\{ \begin{gathered} 0.07\overline{{L_{fi} }}^{0.9} {\text{ for }}\overline{{L_{fi} }} \le {\text{90 (mm) }} \hfill \\ 9.14\sqrt {\frac{{f_{c}{\prime}^{2/3} \times 0.8 \times \overline{{L_{fi} }} }}{{E_{f} \rho_{f} + E_{sw} \rho_{v} }}} {\text{ for }}\overline{{L_{fi} }} {\text{ > 90 (mm)}} \hfill \\ \end{gathered} \right..$$

By taking the equilibrium of the bond force according to the free body diagram (Fig. 2a), the effective (average) strain of the ETS system in the beam can be derived as follows:

20 $$\varepsilon_{fe} = \frac{{\pi d_{f} \tau_{m} \overline{{L_{fi} }} }}{{\pi d_{f}^{2} /4 \times E_{f} }},$$

where *d*_*f*_ is the ETS bar diameter (mm).

The debonding of the ETS-FRP bars to concrete without FRP rupture represents the failure criterion of the ETS-FRP strengthening system in the ETS-FRP-retrofitted beam. Debonding occurs due to crack initiation, opening, and propagation in the beam. According to international specifications (ACI PCR-440.2–17^78^; *fib* 2019^79^), the strain in FRP reinforcement in RC beams is limited by the strain value of 0.004 (concrete integrity) and 0.75*f*_*fu*_/*E*_*f*_ (FRP rupture). According to Eq. (20) and the debonding limit concept, the maximum strain in the ETS-FRP strengthening system in an ETS-strengthened beam can be rewritten as follows:

21 $$A = \min \left( {\frac{{\pi d_{f} \tau_{m} \overline{{L_{fi} }} }}{{\pi d_{f}^{2} /4 \times E_{f} }},\min \left( {0.004,\frac{{0.75f_{fu} }}{{E_{f} }}} \right)} \right).$$

Therefore, the shear resisting force of ETS-FRP strengthening (*V*_*f*_) in the retrofitted beam can be expressed via the bond force of the equivalent pullout scheme.

22 $$V_{f} = N_{f} E_{f} A_{f} A$$

### Calculation procedure

On the basis of the formulations established in “Original 2PKT approach” and “Shear contribution of ETS-FRP strengthening system” sections, the total shear strength (*V*_*total*_) of an ETS-FRP strengthened RC beam can be described by considering the following five shear components:

23 $$V_{total} = V_{clz} + V_{ci} + V_{s} + V_{d} + V_{f} .$$

Apart from the strength caused by the shear components, the total shear capacity can be derived by the moment equilibrium of the tensile force of the bottom flexural reinforcement (*T*), which is expressed as follows:

24a $$V_{T} = T\left( {0.{9}d} \right)/a,$$

24b $$T = E_{s} A_{s} \varepsilon_{t,avg} + \frac{{0.33\sqrt {f{^\prime}_{c} } }}{{\sqrt {1 + 200\varepsilon_{t,avg} } }}A_{c,eff} ,$$

24c $$A_{c,eff} = {\text{min}}\left[ {{2}.{5}\left( {h{-}d} \right)b,bh/{2}} \right],$$

where *a* is the shear span length (mm); *A*_*s*_ is the total area of the steel tension reinforcement (mm^2^); and *A*_*c,eff*_ is the effective area of concrete for the tension stiffening of the longitudinal reinforcement (mm^2^).

**Figure 3:**
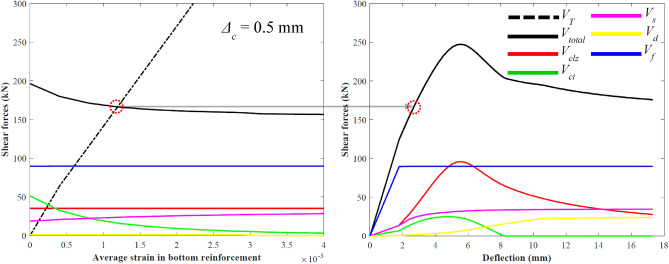
2PKT solution for the full shear load–deflection curve.

The shear strength attributable to the shear components (*V*_*total*_ in Eq. (23)) and section equilibrium (*V*_*T*_ in Eq. (24a)) must be equated. At each step of the displacement of the critical loading zone (*Δ*_*c*_), the shear resisting forces of the shear components and the total shear strength of the ETS-retrofitted beam depend on the average strain in the bottom reinforcement (*ε*_*t,avg*_). The intersection of *V*_*T*_ versus *ε*_*t,avg*_ to *V*_*total*_ versus *ε*_*t,avg*_ is an equilibrium point in the shear load–deflection curve plot of the ETS-strengthened RC beam (Fig. 3). The bisection method is applied to the variable *ε*_*t,avg*_ to find the intersection between *V*_*T*_ and *V*_*total*_. In this manner, the complete shear force–deflection response of the ETS-strengthened beam can be obtained. The iterations for *Δ*_*c*_ and *ε*_*t,avg*_ are needed to determine the steps between load and displacement.

At the point of peak shear force, *V*_*clz*_, *V*_*s*_, *V*_*ci*_, and *V*_*f*_ can reach their own peaks (Fig. 3). Beam failure is attributable to concrete crushing in the compression zone, which can lead to diagonal shear cracking, transverse steel yielding, and FRP debonding.

## Verification of the developed 2PKT approach

### Experimental database

Some experimental programs for investigating the shear behavior of concrete beams strengthened with ETS-FRP bars have been implemented^26–32^. Among the aforementioned studies, the engineering information for the test beams provided by Mofidi et al.^26^, Breveglieri et al.^29^, Bui et al.^30^, and Bui et al.^32^ can conveniently and sufficiently perform 2PKT prediction. In the experiments of Mofidi et al.^26^, Breveglieri et al.^29^, and Bui et al.^30^, the beams have T-shaped sections; meanwhile, rectangular-shaped sections were applied for the beams in the experiments by Bui et al.^32^. The shear span-to-effective depth (*a/d*) ratios for all specimens in the experimental programs of Breveglieri et al.^29^ and Bui et al.^30^ for specimen B1 in the study by Bui et al.^32^ were 2.6, 2.5, and 2.4, which are representative of deep beams. For the remaining strengthened beams, *a/d* ≥ 3.0, which might represent the behavior of the slender beams.

The beams studied by Mofidi et al.^26^ and Breveglieri et al.^29^ adopted CFRP bars for ETS strengthening systems; in their succeeding works^30,32^, the beams were strengthened by ETS-GFRP bars. All tested beams were designed to be dominated by shear failure in the shear zones consisting of ETS-FRP bars. Therefore, the beams were overreinforced with a high amount of longitudinal steel reinforcement (Table 1), inducing shear cracks followed by the yielding of steel stirrups and the debonding of ETS-FRP bars to concrete. The rupture of FRP bars was not detected in those works.

**Table 1:** Beam specifications in available experimental studies.

Study	Beam ID	*b* (mm)	*b*_*f*_ (mm)	*h* (mm)	*d* (mm)	*a/d*	*n*_*b*_	*d*_*s*_ (mm)	*f*_*ys*_ (MPa)	*ρ*_*s*_ (%)	*E*_*s*_ (GPa)	*d*_*sw*_ (mm)	*ρ*_*sw*_ (%)	*E*_*sw*_ (GPa)	*f*_*ysw*_ (MPa)
Mofidi et al.^26^	S0-12d130s	152	508	406	350	3.0	4	25	470	3.69	200	0	–	–	540
	S1-9d260s	152	508	406	350	3.0	4	25	470	3.69	200	8	0.25	200	540
	S1-12d260s	152	508	406	350	3.0	4	25	470	3.69	200	8	0.25	200	540
	S1-12d130s	152	508	406	350	3.0	4	25	470	3.69	200	8	0.38	200	540
	S1-9d260p	152	508	406	350	3.0	4	25	470	3.69	200	8	0.25	200	540
	S3-12d130s	152	508	406	350	3.0	4	25	470	3.69	200	8	0.38	200	540
Breveglieri et al.^29^	2S-C180-90	180	450	400	360	2.5	4	24	598	2.79	200	6	0.10	200	574
	2S-C180-45	180	450	400	360	2.5	4	24	598	2.79	200	6	0.10	200	574
	4S-C180-90	180	450	400	360	2.5	4	24	598	2.79	200	6	0.17	200	574
	4S-C180-45	180	450	400	360	2.5	4	24	598	2.79	200	6	0.17	200	574
Bui et al.^30^	B1	180	450	400	343	2.6	4	25	400	3.18	200	6	0.11	200	240
	B2	180	450	400	343	2.6	4	25	400	3.18	200	9	0.11	200	240
	B3	180	450	400	343	2.6	4	25	400	3.18	200	6	0.24	200	240
	B4	180	450	400	343	2.6	4	25	400	3.18	200	9	0.24	200	240
Bui et al.^32^	B1	150	–	300	250	2.4	4	25	390	5.24	200	9	0.28	200	235
	B2	150	–	300	250	3.6	4	25	390	5.24	200	9	0.28	200	235
	B3	150	–	300	250	4.8	4	25	390	5.24	200	9	0.28	200	235
	B4	150	–	300	250	3.6	4	25	390	5.24	200	9	0.28	200	235
	B5	150	–	300	250	3.6	4	25	390	5.24	200	9	0.28	200	235
	B6	150	–	300	250	3.6	4	25	390	5.24	200	9	0.28	200	235
	B7	150	–	300	250	3.6	4	25	390	5.24	200	9	0.28	200	235
	B8	150	–	300	250	3.6	4	25	390	5.24	200	9	0.28	200	235
	B9	150	–	300	250	3.6	4	25	390	5.24	200	9	0.28	200	235

**Study**	**Beam ID**	***d***_***f***_** (mm)**	***ρ***_***f***_** (%)**	***E***_***f***_** (GPa)**	***f***_***fu***_** (MPa)**		***f***_***c***_***'* (MPa)**	***s***_***m***_** (mm)**	***V***_***total-Exp.***_** (kN)**	***V***_***total-Ana.***_** (kN)**					
Mofidi et al.^26^	S0-12d130s	12.7	0.64	148	1885		25	0.05	180.8	264.13					
	S1-9d260s	9.5	0.18	148	1885		29.6	0.05	260.3	274.3					
	S1-12d260s	12.7	0.32	148	1885		29.6	0.05	266.6	281.7					
	S1-12d130s	12.7	0.64	148	1885		25	0.05	263.6	299.1					
	S1-9d260p	9.5	0.18	155	2800		29.6	0.05	280.7	274.1					
	S3-12d130s	12.7	0.64	148	1885		29.6	0.05	281.8	299.1					
Breveglieri et al.^29^	2S-C180-90	8	0.16	160	1920		32.3	0.05	222.3	247.4					
	2S-C180-45	8	0.22	160	1920		32.3	0.05	320.8	314.3					
	4S-C180-90	8	0.16	160	1920		32.3	0.05	226.1	256.8					
	4S-C180-45	8	0.22	160	1920		32.3	0.05	370.1	334.6					
Bui et al.^30^	B1	10	0.24	50	1076		38	0.12	272.3	272.9					
	B2	10	0.34	50	1076		38	0.12	288.9	290.7					
	B3	10	0.24	50	1076		38	0.12	309.1	282.1					
	B4	10	0.34	50	1076		38	0.12	353.9	300.9					
Bui et al.^32^	B1	8	0.11	46	862		27	0.12	107.0	120.1					
	B2	8	0.11	46	862		27	0.12	99.2	82.7					
	B3	8	0.11	46	862		27	0.12	81.5	65.0					
	B4	8	0.11	46	862		43	0.12	138.0	99.2					
	B5	8	0.11	46	862		27	0.12	89.1	82.7					
	B6	8	0.11	46	862		27	0.12	101.9	82.7					
	B7	8	0.11	46	862		27	0.12	100.2	82.7					
	B8	8	0.11	46	862		27	0.12	92.1	82.7					
	B9	8	0.11	46	862		27	0.12	91.5	82.7					
						Mean of *V*_*total-ana.*_/*V*_*total-exp*_				0.98					
						Coefficient of variation, CoV (%)				16.2					

The effects of the presence of existing stirrups, ETS material types, and percentages and concrete compressive strength on the strengthening efficiency of the ETS-retrofitted beams were also examined in the abovementioned works. The necessary information for those tested beams is summarized in Table 1. The beam shear strength and ETS-FRP shear contribution are also shown in the table. In this study, all beams mentioned in Table 1 were simulated to evaluate the representativeness and accuracy of the 2PKT approach. Then, the simulated 2PKT results were compared with the experimental data.

### Verification

**Figure 4:**
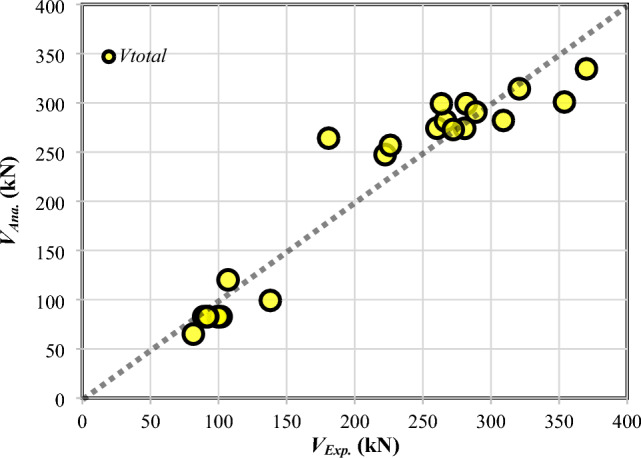
Validation of *V*_*total*_*.*

The results of verification of the shear capacity of the ETS-strengthened beams in the literature^26,29,30,32^ and the use of the developed 2PKT approach to further verify the experimental data are presented in Fig. 4 and Table 1, respectively. The analytical model in this study was developed to predict the shear responses of ETS-FRP-strengthened RC beams. Therefore, only strengthened specimens with ETS-FRP bars from the literature^26,29,30,32^ were used in the model verification. The comparisons between the model calculation and experimental results focus on the total shear strength of the beam (*V*_*total*_). As shown in Table 1, the average of *V*_*total-ana.*_/*V*_*total-exp.*_ is 0.98, and the coefficient of variation (CoV) of the mean is 16.2%. The prominent effects of the variables on the beam shear strength can be well assessed by the 2PKT model. In the studies of Breveglieri et al.^29^ and Bui et al.^30^, the strengthened beams with ETS-FRP bars inclined at 45° or more shear reinforcement had a much greater shear capacity than those with vertical ETS-FRP bars or less-transverse reinforcement. In addition, both 2PKT computation and experiment for the specimens in Bui et al.^32^, compared specimen B4 to other beams, found that the higher the concrete compressive strength was, the larger the total shear strength. These aforementioned findings demonstrate the good agreement and rationale between the developed 2PKT model and the beam shear strength tests. The computation via the developed 2PKT approach for ETS-FRP-strengthened RC beams can also be rapidly implemented.

**Figure 5:**
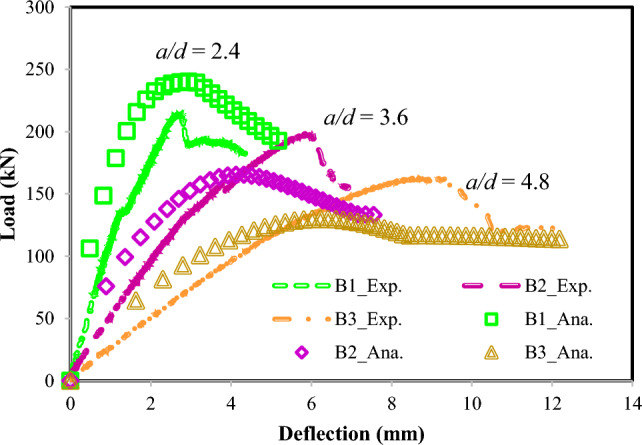
Validation of the experimental results of Bui et al.^32^ for specimens B1, B2, and B3.

The 2PKT model can plot both prepeak and postpeak regimes, which may be necessary for evaluating the ductility displacement properties of ETS-strengthened RC beams. The validation technique applied by 2PKT to test specimens B1, B2, and B3 in the work of Bui et al.^32^ are shown in Fig. 5. The beams obtained *a/d* ratios of 2.4, 3.6, and 4.8 for beams B1, B2, and B3, respectively. Good agreement was established between the experimental and analytical results in the load–displacement curves. Furthermore, the values were consistent in the reduction in the shear resistance and stiffness of the ETS-strengthened RC beams with increasing *a/d* ratios. These findings can be explained by the behavior of beams with large *a/d* ratios during beam action and their shear actions decreasing or even becoming negligible. Therefore, the shear resisting forces caused by the presence of concrete are significantly reduced with increasing *a/d* ratios, a relationship that governs the entire strengthened beam capacities.

**Figure 6:**
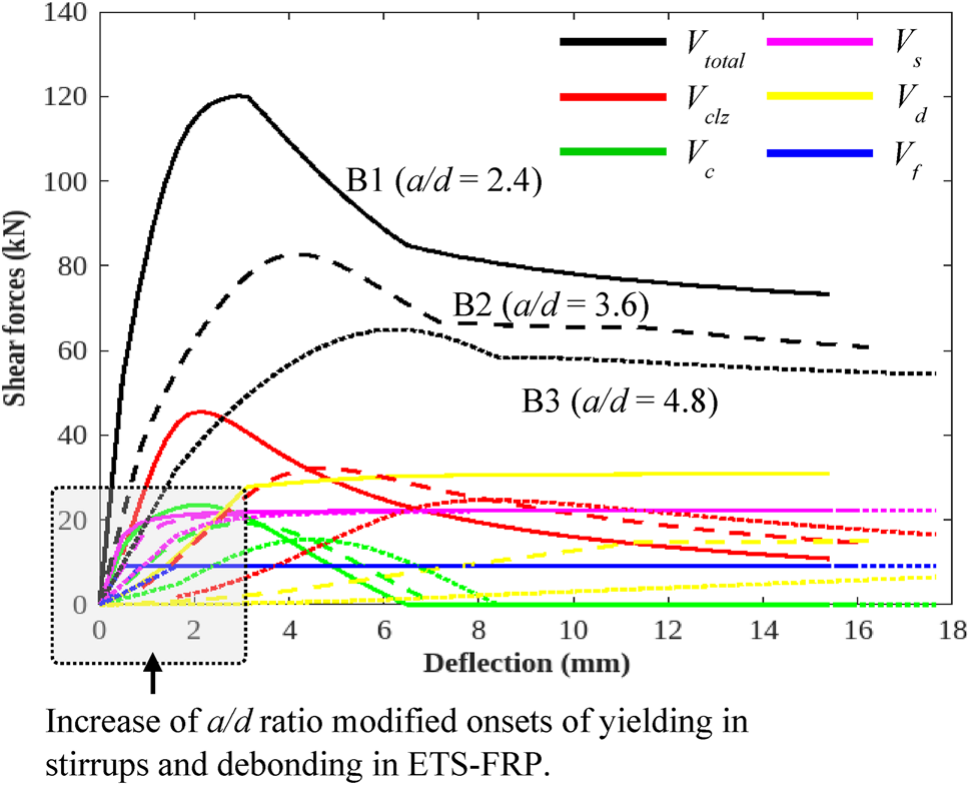
2PKT analyses for specimens B1, B2, and B3 in the study of Bui et al.^32^.

The abovementioned result is confirmed by the 2PKT analyses of the specimens shown in Fig. 6, in which the reduction in concrete shear strength relating to the *a/d* ratio is evident by the reductions in *V*_*clz*_ and *V*_*ci*_. At *a/d* = 2.4 (usually categorized as a deep beam), *V*_*clz*_ primarily governs the failure of beam B1, a situation that agrees well with the test monitoring. Computation via the 2PKT approach is shown in Fig. 6. The increasing *a/d* ratios of the ETS-strengthened beams modified the onsets of stirrup yielding and ETS-FRP debonding with large deflections. Although no clear experimental evidence for those observations was discovered, the beam with a high *a/d* ratio would cause an early trigger of the bending mechanism but would later activate the shear resisting mechanism. Otherwise, the maximum shear contribution for each component (i.e., stirrups and ETS) would be unchanged under the effect of the *a/d* ratio. Here, the 2PKT analyses utilized a set of failure criteria for the transverse steels and ETS-FRP strengthening system in an ETS-strengthened beam representing the yielding of the whole stirrups and debonding of ETS-FRP elements.

The reliability of the developed 2PKT model for predicting the shear capacity of ETS-FRP-strengthened RC beams can be elucidated above. Another benefit of the developed 2PKT model is that it promptly produces the calculation output only after a few seconds. This feature can help users generate a large range of data for ETS-strengthened beams without using high-performance and expensive computational tools. “Machine learning approach” section will focus on the implementation of the ML models based on the data generated by the 2PKT formulations to predict the shear capacity of ETS-FRP-strengthened beams.

## Machine learning approach

The ML domain focuses on the utilization of data to enhance performance in various tasks, such as clustering, classification, and regression. In regression tasks, the algorithm predicts outcomes based on the relationship between features and a target variable. Several ML algorithms are currently used for regression, such as linear regression, lasso, decision tree, random forest, support vector regression, gradient boost, and artificial neural networks (ANNs). In this study, four widely used algorithms, which are known for their efficiency and high accuracy, are utilized: random forest, extreme gradient boosting (XGBoost), light gradient boosting machine (LightGBM), and ANN. In this manner, the shear capacity of ETS-FRP-strengthened beams can be predicted using the 2PKT approach.

### Machine learning models

**Figure 7:**
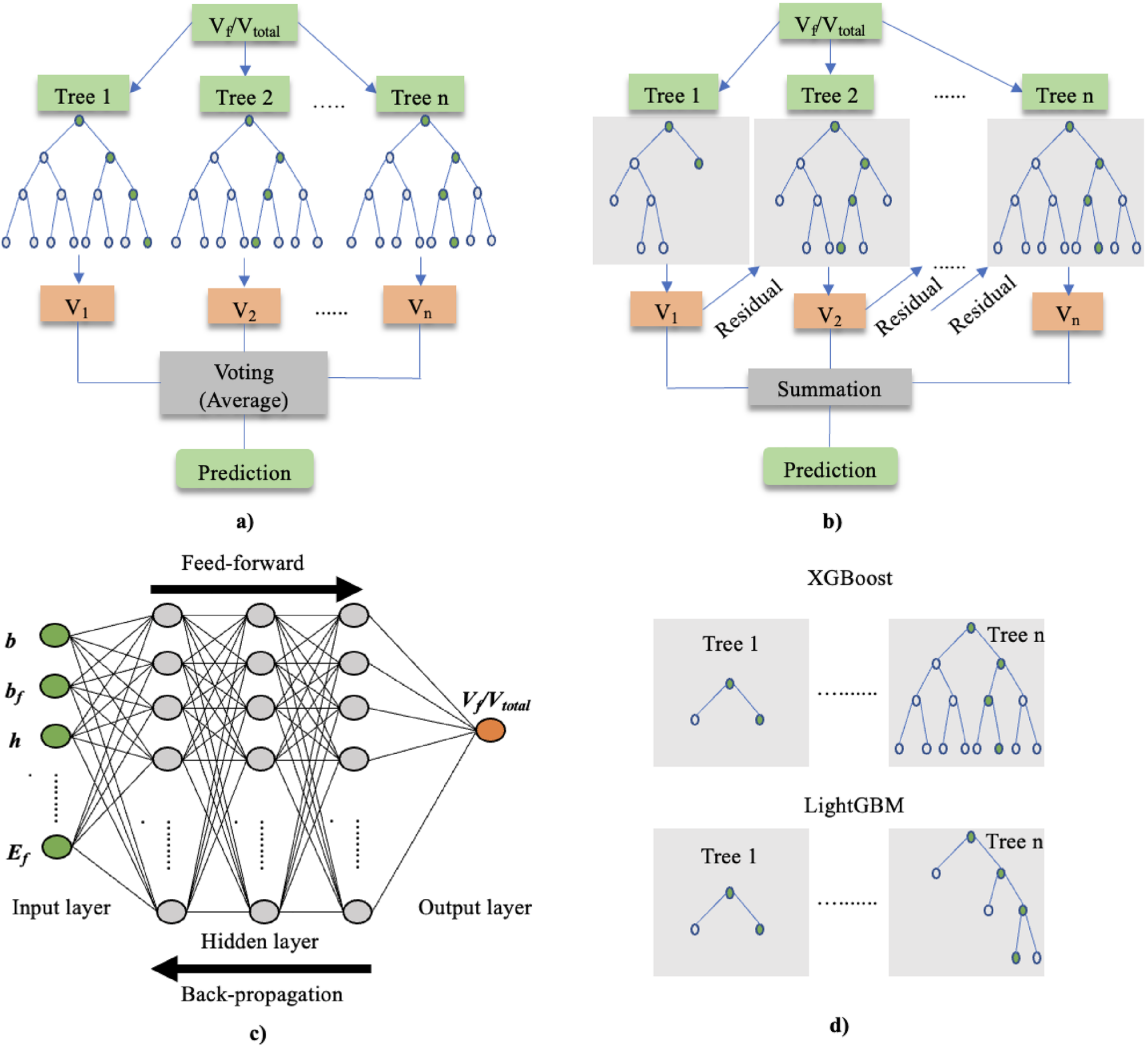
ML algorithms: (**a**) random forest; (**b**) XGBoost and LightGBM; (**c**) ANN; (**d**) difference between XGBoost and LightGBM.

Random forest^80^ is a popular ensemble technique that uses bootstrapping and random feature selection to create several decision trees. The trees are uncorrelated, and their predictions are merged using a voting process to obtain the result (Fig. 7a). XGBoost^81^ is built on a gradient-boosting framework that sequentially trains multiple decision trees, with each tree trained to correct the errors of the previous tree. The architecture of the XGBoost algorithm is shown in Fig. 7b. LightGBM^82^ shares many similarities with XGBoost but grows tree leafwise instead of depthwise (Fig. 7d), resulting in a much faster and more memory-efficient training. XGBoost and LightGBM are highly effective and widely used in industry and academia.

ANNs are complex systems composed of three fundamental layers: the input, hidden, and output layers. The input layer receives raw data and passes these data to the hidden layer. The hidden layer is the computational core of the network, and its neurons perform complex computations on the input data. The output layer combines input and hidden information to generate an output value from which predictions for the response variables are provided. ANNs are a powerful tool for data analysis, and understanding the fundamental layers is necessary in unlocking the full potential of this algorithm^83^.

Furthermore, the ANN model includes feedforward and backward propagations. Feedforwarding is the process of inputting data into a neural network and processing them layer by layer through the network until a prediction is generated. In the feedforward process, each neuron in the network receives input from the neurons in the previous layer, performs a computation, and passes the output to the neurons in the next layer. This process continues until the output layer is reached and the final prediction is generated. Backward propagation is the process of calculating the gradient of the loss function concerning the weights and biases of the neural network. The gradient updates the weights and biases during training, aiming to minimize the loss function and improve the network’s accuracy. Figure 7c shows an example of ANN architectures. The selected studies^80–83^ can be referenced for additional details about the aforementioned algorithms.

### Dataset preparation

#### Data collection

After confirming the excellent performance of the developed 2PKT via experimental validation, this method was used to simulate more than 2643 data points, encompassing all feasible and realistic variable scenarios. Then, this dataset was utilized to implement ML models to predict the shear strength of ETS-strengthened RC beams. During the simulation, the technical constraints regarding the mechanics and details of the reinforcement were considered to ensure realistic and meaningful data. The constraints include the following conditions:

25 $$100 \times \frac{{\left| {V_{total} - V_{T} } \right|}}{{V_{total} }} < 0.1,$$

26 $$0.75f_{fu} /E_{f} > 0.004,$$

27 $${\text{s}}_{sw} { } \le \frac{a}{2},$$

28 $${\text{s}}_{f} { } \le \frac{a}{2},$$

29 $$\frac{{{\text{n}}_{b} {\text{d}}_{b} }}{2} + 30 \times \left( {\frac{{{\text{n}}_{b} }}{2} - 1} \right) + 50 \le \frac{h}{2},$$

30 $$2 \times \left( {{\text{d}}_{b} + {\text{ d}}_{sw} } \right) + 120 \le {\text{b}}{.}$$

The condition in Eq. (25) represents the force equilibrium between shear forces derived by shear components (*V*_*total*_) and flexural moment (*V*_*T*_); it is the termination condition of the computation. The condition in Eq. (26) considers the debonding failure criteria of the ETS-FRP bonded to concrete based on the strain limit of 0.004 (*ε*); that is, no rupture of FRP is examined in the model computation. This condition of FRP debonding from concrete, which was observed in past studies (i.e., pertaining to the experimental tests of ETS-FRP-strengthened RC beams), is safe and common for design practice. The conditions in Eqs. (27) and (28) involve the allowable spacing of the existing steel stirrups (*s*_*sw*_) and ETS-FRP strengthening bars (*s*_*f*_), which is smaller than half of the shear span (*a*). Equations (29) and (30) satisfy the detailed conditions of the steel and ETS-FRP reinforcement located in the beam section (width and height directions).

#### Data description

After collecting the simulation data, the relationship between the independent and target variables needed to be determined before experimenting with any ML algorithm. Correlation can be employed for bivariate analysis, i.e., measuring the relationship between two variables. The measure of correlation is called the correlation coefficient. Pearson’s correlation coefficient is determined by linear association. The correlation coefficient value (*R*) ranges from − 1 to + 1, where − 1 indicates a negative correlation, + 1 signifies a positive correlation, and 0 denotes the absence of a correlation between two variables. Equation (31) shows the formulation of Pearson’s correlation coefficient, in which the covariance ratio between two variables (*S*_*xy*_) is divided by their standard deviation (*S*_*x*_, *S*_*y*_).

**Figure 8:**
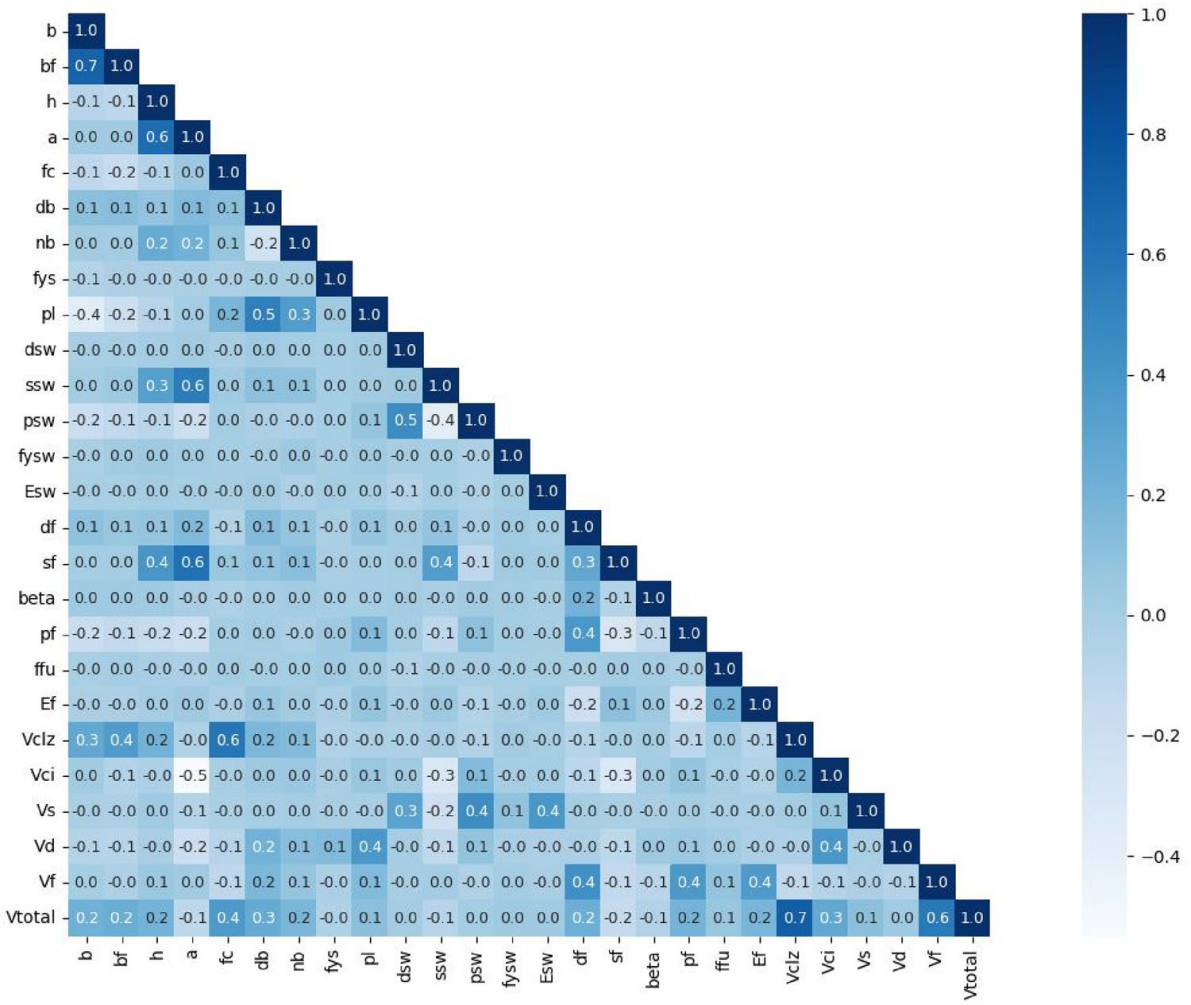
Correlations between variables.

Figure 8 illustrates the pairwise correlation coefficient between the variables used for analysis. The variables *f’*_*c*_, *ρ*_*f*_, *d*_*f*_, *E*_*f*_, *s*_*f*_, and *β* have considerable effects on the shear contribution of the ETS-FRP strengthening system (*V*_*f*_). The aforementioned variables govern the amounts and properties of the ETS-FRP strengthening bars or the bond shear stress between ETS-FRP bars and concrete. Meanwhile, the variables *d*_*b*_, *n*_*b*_, and *ρ*_*l*_ affect the shear strength caused by bending (*V*_*T*_), consequently modifying *V*_*f*_ via force equilibrium. Most variables influence the total shear strength of ETS-strengthened RC beams (*V*_*total*_) caused by the interrelationships among parameters in the formulations of the shear components.

31 $$R = \frac{{S_{xy} }}{{S_{x} S_{y} }}$$

**Figure 9:**
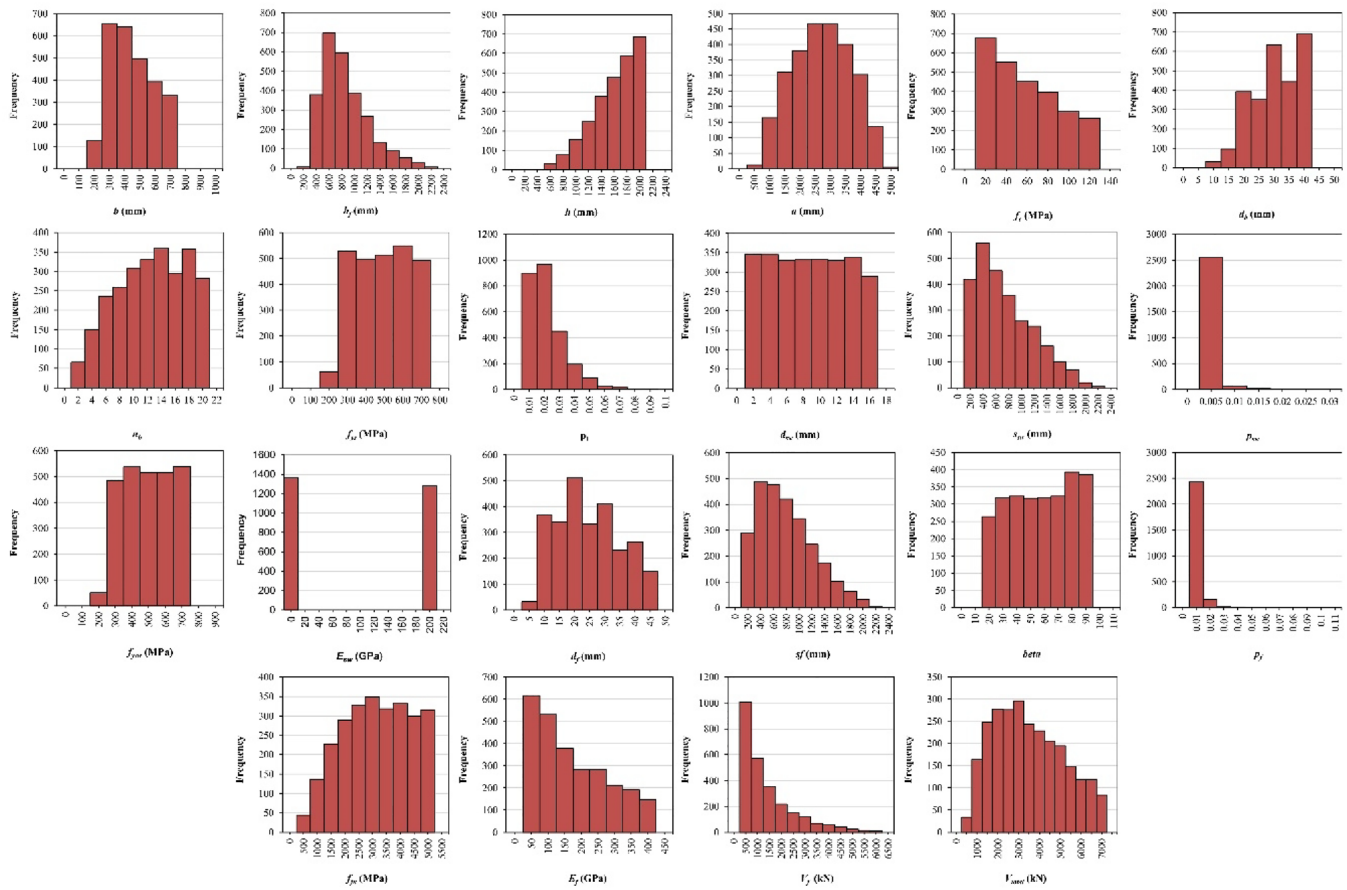
Histogram of variables.

According to the data presented in Table 2, the original input parameters exhibit differences in scale. When the input variables have different scales, the ML algorithms tend to give more weight to the variables with larger scales, which leads to biased and incorrect predictions apart from much slower convergence and poor performance of the model^84^. Therefore, the data need to be normalized to overcome the aforementioned issues. In this study, the max–min normalization method was adopted to normalize all variables in the dataset to a range from 0 to 1 by using Eq. (32). Figure 9 shows the data distribution for each variable.

32 $$x_{norm} { } = { }\frac{{x - { }x_{min} }}{{x_{max} - { }x_{min} }}$$

**Table 2:** Feature description of ETS-FRP-strengthened beams.

Variables	Unit	Minimum	Maximum	Mean	Standard deviation
*b*	mm	150	700	408	145
*b*_*f*_	mm	150	2100	754	362
*h*	mm	340	2000	1521	354
*a*	mm	270	4540	2481	953
*f*_*c*_	MPa	5	120	51	34
*d*_*b*_	mm	8	40	29	8
*n*_*b*_	No unit	2	20	13	5
*f*_*ys*_	MPa	200	700	449	148
*p*_*l*_	No unit	0.0006	0.085	0.016	0.012
*d*_*sw*_	mm	1	15	7.83	4.55
*s*_*sw*_	mm	50	2160	650	444
*ρ*_*sw*_	No unit	1.13E-06	0.0253	0.0010	0.0019
*f*_*ysw*_	MPa	200	700	454	147
*E*_*sw*_	mm	0	200	97	100
*d*_*f*_	mm	4	44	23	10
*s*_*f*_	mm	50	2140	705	436
*β* (or beta)	No unit	15	90	55	23
*ρ*_*f*_	No unit	0.0003	0.0993	0.0039	0.0061
*f*_*fu*_	MPa	200	5000	2905	1230
*E*_*f*_	GPa	10	400	152	110
*V*_*f*_	kN	6	5930	1136	1138
*V*_*total*_	kN	142	6998	3254	1674

**Figure 10:**
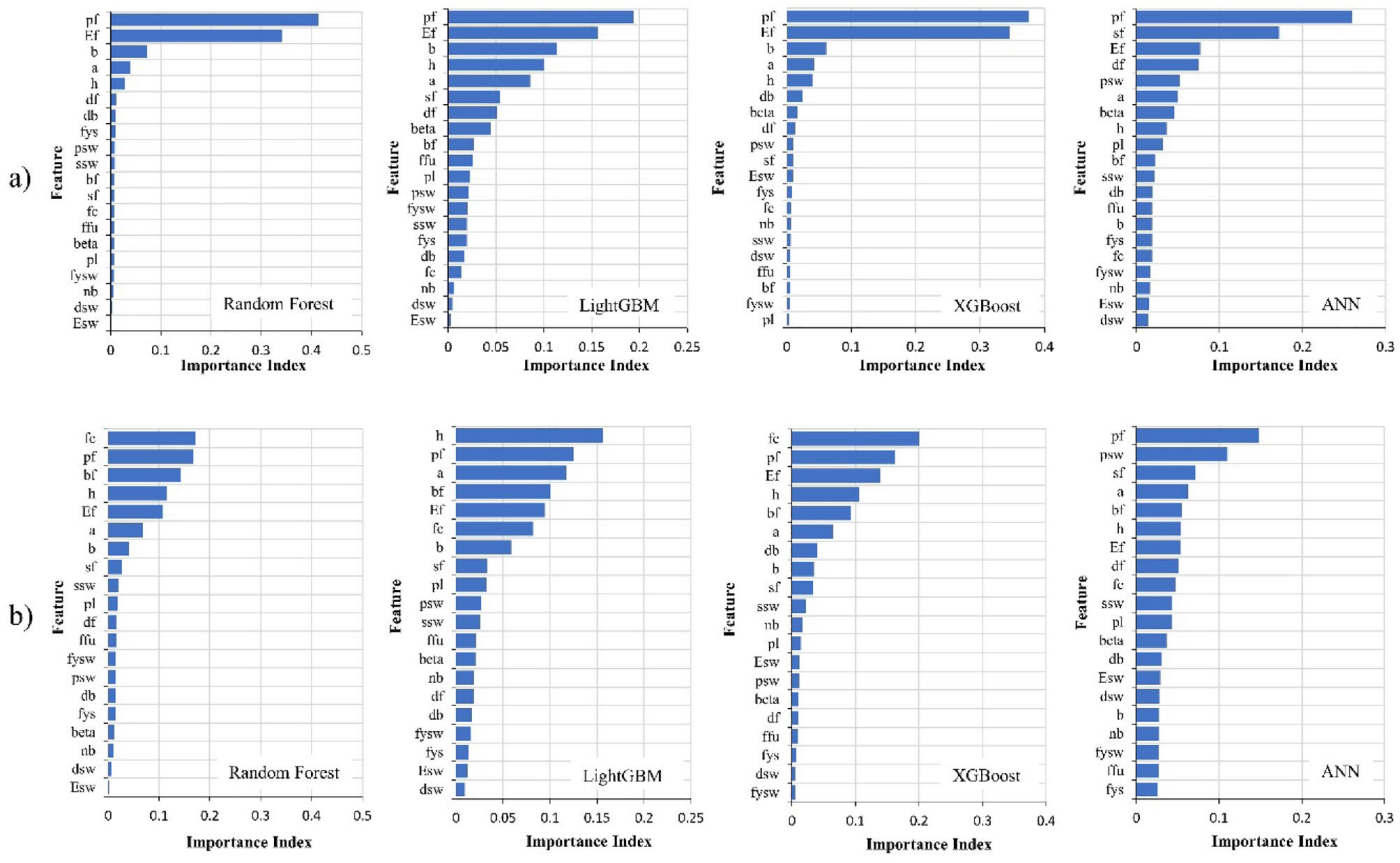
Feature importance corresponding to (**a**) *V*_*f*_ and (**b**) *V*_*total*_*.*

Figure 10 shows the feature importance corresponding to *V*_*f*_ and *V*_*total*_. The method of calculating feature importance varies depending on the ML algorithms, and the analyzed feature importance differs from each algorithm. In general, the analysis reveals that *p*_*f*_, *E*_*f*_, *s*_*f*_, *b*, *a*, and *h* are the most important features for predicting *V*_*f*_ and *V*_*total*_. This outcome is reasonable from an engineering perspective.

#### Evaluation metrics

In this study, four metrics, including mean absolute error (MAE), mean squared error (MSE), root mean square error (RMSE), and coefficient of determination (*R*^2^), were used to evaluate the performance of the ML models. The mathematical expressions and descriptions of these metrics are presented in Table 3.

**Table 3:** Performance evaluation metrics for ML models.

Equation	Description
$MAE = \frac{1}{n}\mathop \sum \limits_{i = 1}^{n} \left| {x_{i} - \hat{x}_{i} } \right|$	Average of the absolute difference between the actual values and the predicted values
$MSE = \frac{1}{n}\mathop \sum \limits_{i = 1}^{n} (x_{i} - \hat{x}_{i} )^{2}$	Used to measure the average squared difference between the actual values and the predicted values
$RMSE = \sqrt {\frac{{\mathop \sum \nolimits_{i = 1}^{N} \left( {x_{i} - \hat{x}_{i} } \right)^{2} }}{N}}$	It measures the average magnitude of the error in the predicted values
$R^{2} = 1 - \frac{{SS_{res} }}{{SS_{tot} }}$	Presents the proportion of the variance in the dependent variable explained by the independent variables

#### Implementing the ML algorithms

Several hyperparameters can be used in ML algorithm operations. However, the meaning of these hyperparameters and how they can be optimized to effectively design and train ML models must be understood. In random forest, XGBoost, or LightBoost, the hyperparameter “n_estimators” determines the number of decision trees in each ensemble. Increasing the number of estimators can improve performance up to a certain point, but several trees may lead to overfitting and increased computational cost. The “max_depth” hyperparameter controls the maximum depth of each decision tree in the ensemble, and limiting it can prevent overfitting and improve model generalization. In a random forest algorithm, other hyperparameters can be tuned to control overfitting and improve the generalization ability of models. “Min_samples_split” specifies the minimum number of samples required to split a node in a decision tree, which helps in generalization by enabling the trees to be less prone to overfitting. Similarly, “min_samples_leaf” sets the minimum number of samples needed to be in a leaf node of a decision tree, thus controlling overfitting. “Max_features” controls the maximum number of features to be considered when splitting a node and plays a crucial role in introducing randomness and diversity into the trees. “Max_samples” specifies the maximum number of samples used for training each decision tree, which can be set as a fixed number or as a fraction of the total number of samples.

In XGBoost, critical hyperparameters shape the modeling behavior, such as the learning rate (eta), min_child_weight, gamma, subsample, colsample_bytree, reg_alpha, and reg_lambda. The learning rate dictates the training step size, while min_child_weight enforces a minimum data sum needed for node splits, thus guarding against overfitting. Gamma contributes to regularization by setting the minimum loss reduction for splits, and subsample introduces randomness by selecting a data subset. Colsample_bytree randomly picks a fraction of features for each tree. Reg_alpha and reg_lambda provide L1 and L2 regularization, enhancing model stability. LightGBM shares some hyperparameters in cases where the learning rate and colsample_bytree align with XGBoost. In LightGBM, bagging_fraction (subsample) augments generalization by sampling data in boosting rounds, and feature_fraction diversifies models by selecting random feature subsets. The num_leaves parameter controls tree complexity and interpretability. Properly configuring these hyperparameters is pivotal for maximizing the performance of both XGBoost and LightGBM across a broad spectrum of ML tasks.

Several critical hyperparameters in an ANN affect its architecture and training process. The n_layers or number of layers define the network depth and complexity, with the input, hidden, and output layers contributing to the network structure. The learning rate controls the step size during training, influencing the convergence speed and stability. Activation functions introduce nonlinearity, enabling the network to learn complex patterns. Batch size determines the number of examples processed in each training step, affecting memory usage and efficiency. Neurons or units in each layer determine the capacity of the network to represent data. Epochs specify the number of passes through the entire training dataset. Dropout is a regularization technique that randomly deactivates neurons during training, mitigating overfitting. The dropout rate sets the probability of neuron deactivation, fine-tuning the regularization effect.

As mentioned above, hyperparameters play a key role in ensuring the good performance of each ML model. ML algorithms often require the fine-tuning of various hyperparameters, which are unique to each problem. The wide range of hyperparameters, coupled with the need to find the best possible combination, makes it impossible to cover all scenarios. Thus, the present study employed HyperOpt, a tool designed to automate the search for optimal hyperparameter configurations. Then, Bayesian optimization was utilized and reinforced by the sequential model-based global optimization methodology^85^.

The hyperparameter optimization process involves the following steps: first, a surrogate model of the objective function is constructed using data from past evaluations. Second, this model is used to identify hyperparameters that can yield the best performance. Third, the selected hyperparameters are tested on the actual objective function by training the model and evaluating its performance metric. The results obtained in this step are used to update the surrogate model (i.e., the fourth step). Steps 2 to 4 are repeated iteratively, often with a maximum iteration or time constraint. Finally, the best-performing hyperparameters across all trials are selected as the optimal configuration^85^.

**Figure 11:**
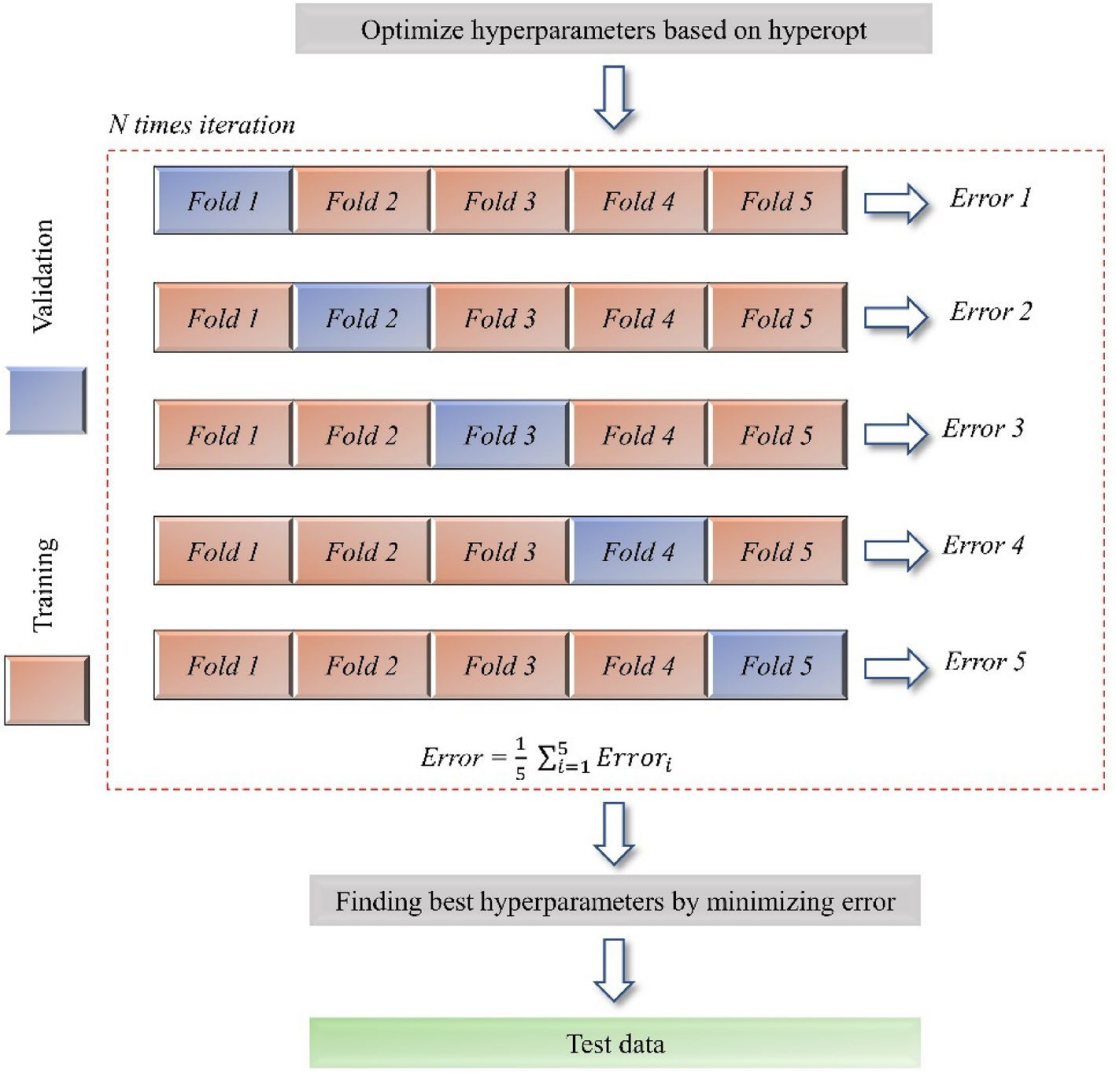
Process of implementing ML algorithms.

Additionally, k-fold cross-validation was implemented to prevent overfitting and ensure that the trained model is reliable for real-world applications^86^. Figure 11 illustrates the process of implementing the ML algorithms, which were applied to four different models to compare their performance in predicting the shear strength of ETS-FRP-strengthened beams. The dataset was split into two subsets, with 80% of the data used for training and the remaining 20% for testing. In the training process, the data were also split into 80% for training and 20% for validation by using fivefold cross-validation. Subsequently, the optimal hyperparameters for each ML algorithm were obtained (Table 4).

**Table 4:** Hyperparameter optimization of the four ML models.

Method	Range of hyperparameters	Optimum hyperparameters for *V*_*f*_	Optimum hyperparameters for *V*_*total*_
Random Forest	+ n_estimators = [50, 1000] + max_depth = [1,50] + min_samples_split = [2,20] + min_samples_leaf = ^2,20^ + max_features = [auto, sqrt, log2, none, 0.1, 0.2, 0.3, 0.4, 0.5] + max_samples = [0.1, 1]	+ n_estimators = 730 + max_depth = 20 + min_samples_split = 12 + min_samples_leaf = 7 + max_features = auto + max_samples = 0.85	+ n_estimators = 640 + max_depth = 31 + min_samples_split = 2 + min_samples_leaf = 5 + max_features = none + max_samples = 0.96
XGBoost	+ n_estimators = [50, 1000] + learning rate = [0.001, 0.3] + max_depth = [3,10] + min_child_weight = [1,20] + gamma = [0, 10] + subsample = [0.5, 1] + colsample_bytree = [0.5, 1] + reg_alpha = [0, 1] + reg_lamda = [0, 1]	+ n_estimators = 650 + learning rate = 0.19 + max_depth = 1 + min_child_weight = 12 + gamma = 0.0063 + subsample = 0.54 + colsample_bytree = 0.70 + reg_alpha = 0.87 + reg_lamda = 0.68	+ n_estimators = 790 + learning rate = 0.13 + max_depth = 2 + min_child_weight = 4 + gamma = 0.022 + subsample = 0.94 + colsample_bytree = 0.61 + reg_alpha = 0.69 + reg_lamda = 0.086
LightGBM	+ n_estimators = [50, 2000] + learning rate = [0.001, 0.3] + max_depth = [3,10] + bagging_fraction = [0, 1] + feature_fraction = [0, 1] + num_leaves = [20, 3000] + colsample_bytree = [0.5, 1]	+ n_estimators = 800 + learning rate = 0.1 + max_depth = 3 + bagging_fraction = 0.17 + feature_fraction = 0.86 + num_leaves = 2660 + colsample_bytree = 0.99	+ n_estimators = 1000 + learning rate = 0.11 + max_depth = 3 + bagging_fraction = 0.89 + feature_fraction = 0.89 + num_leaves = 2460 + colsample_bytree = 0.89
ANN	+ n_layer = [1,10] + learning rate = [0.0001, 0.1], + activation = [elu, relu] + batch_size = [16, 32, 64, 128, 256] + neurons = [10, 300] + epochs = [50, 500] + dropout = [False, True] + dropout_rate = [0.1, 0.5]	+ n_layer = 2 + learning rate = 0.06 + activation = elu, + batch_size = 128 + neurons = 100 + epochs = 150 + dropout = False + dropout_rate = None	+ n_layer = 4 + learning rate = 0.008 + activation = relu + batch_size = 256 + neurons = 200 + epochs = 200 + dropout = False + dropout_rate = None

#### Results and analysis

The performance of the proposed approach was comprehensively evaluated in the present study. In particular, the results derived by the hybrid 2PKT–ML model were compared with those calculated by the shear design models provided in the ACI^78^ and JSCE^87^ guidelines. The equations for computing the strengths provided by the shear components of ETS-FRP-strengthened RC beams employing the ACI guidelines^78^ are expressed as follows:

33a $$V_{c} = 0.23\sqrt {f{^\prime}_{c}} bd,$$

33b $$V_{sw} = A_{sw} f_{ysw} \frac{{d\left( {\cot \theta + \cot \beta } \right)}}{{s_{sw} }}\sin \beta ,$$

33c $$V_{f} = A_{f} E_{f} \varepsilon_{fe} \frac{{h\left( {\cot \theta + \cot \beta } \right)}}{{s_{f} }}\sin \beta ,$$

33d $$\varepsilon_{fe} = {\text{min (0}}{.004, 0}{\text{.75}}\varepsilon_{fu} {)}{\text{.}}$$

The JSCE guideline^87^ stipulates the following expressions for the shear components:

34a $${\text{V}}_{{\text{c}}} = 0.2\sqrt[3]{{{\text{f}}_{{\text{c}}}{\prime} }}\sqrt[4]{{\frac{1000}{{\text{d}}}}}\sqrt[3]{{100{\uprho }_{s} }}\left( {0.75 + \frac{1.4}{{\text{a/d}}}} \right){\text{bd,}}$$

34b $$V_{sw} = A_{sw} f_{ysw} \frac{{7d\left( {\cot \theta + \cot \beta } \right)}}{{8s_{sw} }}\sin \beta ,$$

34c $$V_{f} = A_{f} E_{f} \varepsilon_{fe} \frac{{h\left( {\cot \theta + \cot \beta } \right)}}{{s_{f} }}\sin \beta ,$$

34d $$\varepsilon_{fe} = \sqrt {\left( {\frac{h}{0.3}} \right)^{ - 0.1} f{^\prime} _{c}\frac{{\rho_{s} E_{s} }}{{\rho_{f} E_{f} }}} \times 10^{ - 4} .$$

where *A*_*sw*_ is the cross-sectional area of the two-leg stirrup (mm^2^); *A*_*f*_ is the cross-sectional area of a single ETS-FRP bar (mm^2^); *V*_*c*_ is the concrete shear strength (kN); *V*_*sw*_ is the shear resistance by steel stirrups (kN); *V*_*f*_ is the ETS-FRP shear contribution (kN);* ε*_*fe*_ is the effective strain in the ETS-FRP strengthening system; *ε*_*fu*_ = *f*_*fu*_/*E*_*f*_ is the rupture strain of the FRP bar; *ρ*_*s*_ is the longitudinal steel reinforcement ratio; *f*_*ysw*_ is the yielding strength of steel stirrups (MPa); *s*_*sw*_ is the transverse steel spacing (mm); *s*_*f*_ is the ETS-FRP bar spacing (mm); *θ* is the shear crack angle taken as 45° for all specimens (°); and *β* is the inclination of ETS strengthening bars (°).

**Figure 12:**
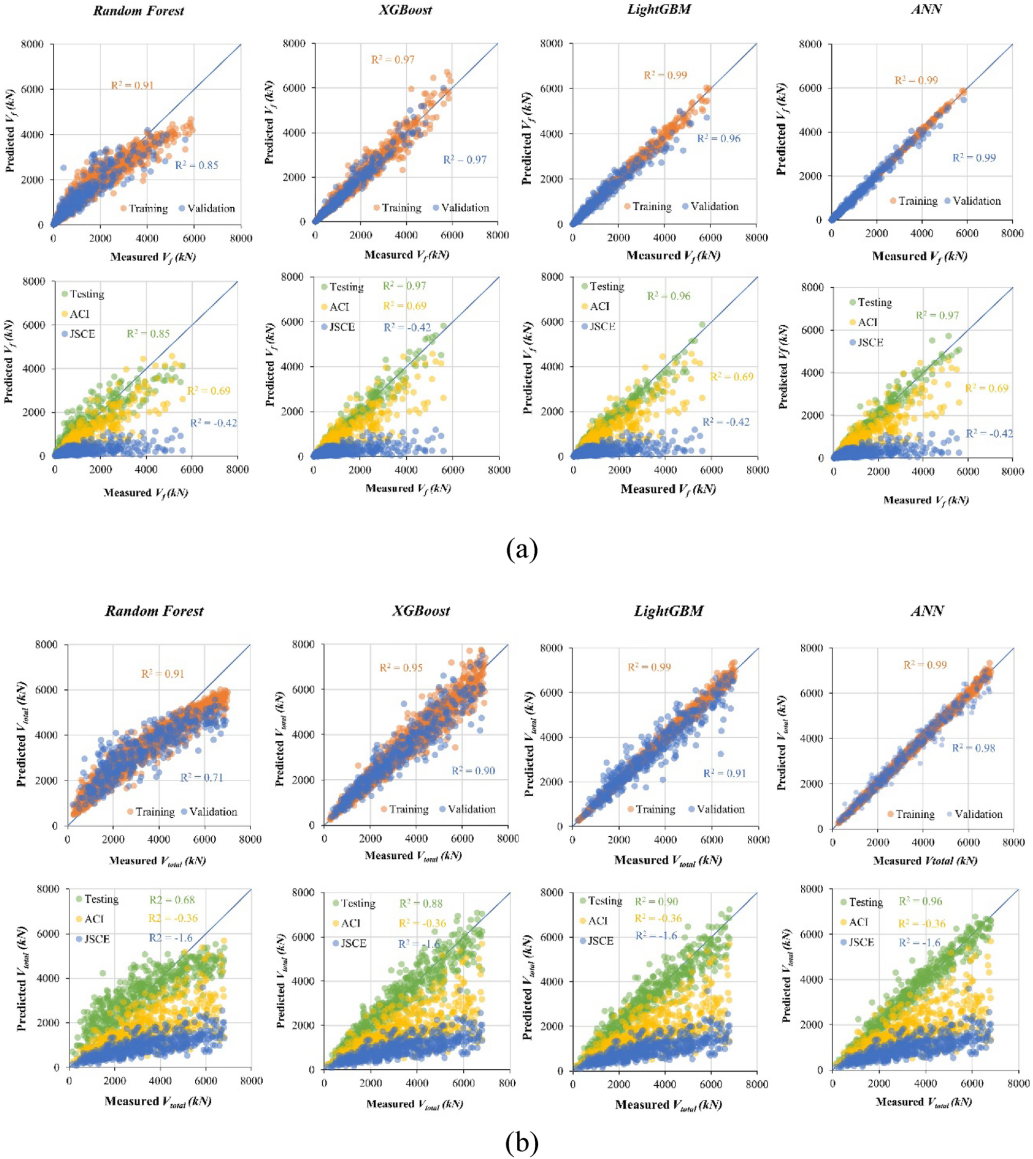
Comparisons of the four ML algorithms in predicting (**a**) *V*_*f*_ and (**b**) *V*_*total*_*.*

Figures 12a and b show the results of the regression analysis for the four ML algorithms used to predict *V*_*f*_ and *V*_*total*_, respectively, after training, validation, and testing. *V*_*f*_ is strongly correlated with *p*_*f*_, *E*_*f*_, *d*_*f*_, *a*, *b*, and *h*. Here, *V*_*total*_ is the sum of *V*_*clz*_, *V*_*ci*_, *V*_*s*_, and *V*_*d*_, which complicates the establishment of a relationship between *V*_*total*_ and the variables by the ML algorithms. As a result, the performance of the ML algorithms in predicting *V*_*total*_ is worse than that for *V*_*f*_.

Among the various algorithms employed in this work, the random forest algorithm exhibited poor predictive capabilities for both *V*_*f*_ and *V*_*total*_ in the training, validation, and testing phases. For *V*_*f*_ prediction, the random forest algorithm achieved R-squared (R^2^) values of 0.91, 0.85, and 0.85 for the training, validation, and testing datasets, respectively. In the case of *V*_*total*_ prediction, the random forest algorithm similarly underperformed, yielding R^2^ values of 0.91, 0.85, and 0.85 for the training, validation, and testing datasets, respectively.

The XGBoost, LightGBM, and ANN algorithms consistently demonstrated predictive solid performance for *V*_*f*_ and *V*_*total*_. Regarding *V*_*f*_ prediction, XGBoost exhibited exceptional accuracy across all phases, boasting R^2^ values of 0.97 for the training, validation, and testing datasets. However, XGBoost proved less suitable for *V*_*total*_ prediction, as its R^2^ values were 0.95, 0.90, and 0.88 for the training, validation, and testing datasets, respectively. The performance of LightGBM was close to that of XGBoost.

The deep network architecture of the ANN model proved to be the top performer, particularly when forecasting *V*_*total*_. Its R^2^ values of 0.99, 0.98, and 0.96 for the training, validation, and testing phases, respectively, were remarkable. Furthermore, during the training and validation phases, the ANN model continued to outperform XGBoost and LightGBM in predicting *V*_*f*_. In the testing phase, the performance of the ANN model was on par with that of XGBoost and LightGBM. The ANN model could predict *V*_*f*_ with R^2^ values of 0.99, 0.99, and 0.97 for the training, validation, and testing datasets, respectively. These findings showcase the capability of the ANN model to manage intricate challenges, as evidenced by its superior *V*_*total*_ forecasting performance. Overall, on the basis of the R^2^ values (Fig. 12a and b), the performance of all the proposed ML models in predicting *V*_*f*_ and *V*_*total*_ was greater than that of the existing ACI^78^ and JSCE^87^ models.

**Figure 13:**
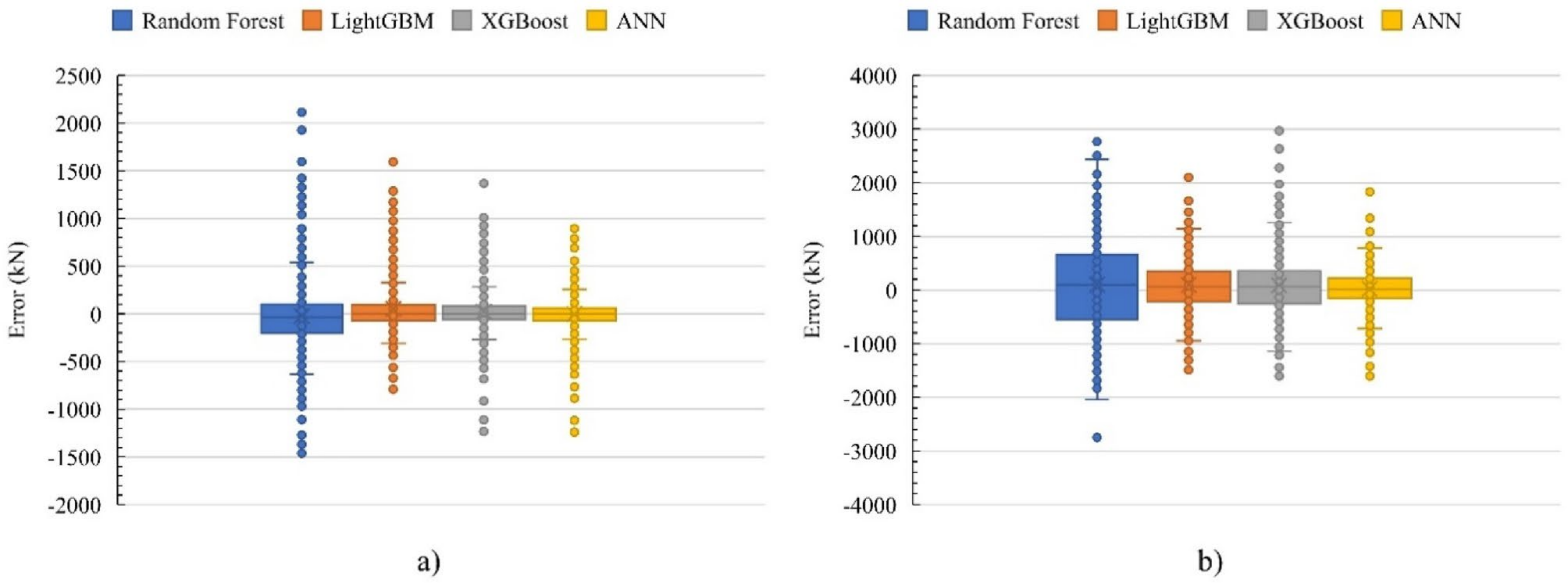
Error of ML algorithms in predicting (**a**) *V*_*f*_ and (**b**) *V*_*total*_*.*

Table 5 provides a summary of the performance of the four algorithms across five evaluation metrics. ANN outperformed the other algorithms in the training and validation steps for predicting both *V*_*f*_ and *V*_*total*_. However, XGBoost slightly outperformed the ANN in predicting *V*_*f*_ with the testing dataset in other metrics, such as MAE, MSE, and RMSE, although the differences were not statistically significant. XGBoost and ANN shared an R^2^ value of 0.97. In terms of forecasting *V*_*total*_, ANN consistently outperformed the alternatives. Figure 13 presents the errors of each algorithm in predicting *V*_*f*_ and *V*_*total*_. ANN obtained the best performance in predicting the shear strength of the ETS-strengthened beams, as indicated by its lower error and less variation compared with those of the other three algorithms. Overall, the findings demonstrate that the ANN model is stable in calculating the beam shear strength and ETS-FRP shear contribution.

**Table 5:** Comparison of the performance of ML algorithms in predicting the shear strengths of ETS-strengthened beams.

ML Algorithm	Metric											
	MAE	MSE	RMSE	R^2^	MAE	MSE	RMSE	R^2^	MAE	MSE	RMSE	R^2^
	Training				Validation				Testing			
	*V*_*f*_											
Random Forest	202.9	114,657	338.6	0.91	267.7	179,715	423.9	0.85	277.5	188,428	434.1	0.85
XGBoost	109.3	38,110	195.2	0.97	114.7	35,687	188.9	0.97	**117.4**	**40,823**	**202.0**	**0.97**
LightGBM	56.1	9108	95.4	0.99	134.9	50,948	225.7	0.96	134.3	49,349	222.1	0.96
ANN	**23.6**	**1172**	**34.2**	**0.99**	**77.4**	**14,032**	**118.0**	**0.99**	123.4	41,122	203.0	**0.97**
	*V*_*total*_											
Random Forest	439.6	317,943	563.9	0.89	703.5	807,784	898.8	0.71	753.9	884,689	940.6	0.68
XGBoost	257.9	129,387	359.7	0.95	390.9	291,825	540.2	0.90	419.1	326,527	571.4	0.88
LightGBM	94.4	17,429	132.0	0.99	360.3	253,794	503.8	0.91	381.3	266,543	516.3	0.90
ANN	**75.3**	**9943**	**99.7**	**0.99**	**138.6**	**35,733**	**189.0**	**0.99**	**255.3**	**122,574**	**350.0**	**0.96**

Bold values indicate the best metric values of four ML networks.

**Figure 14:**
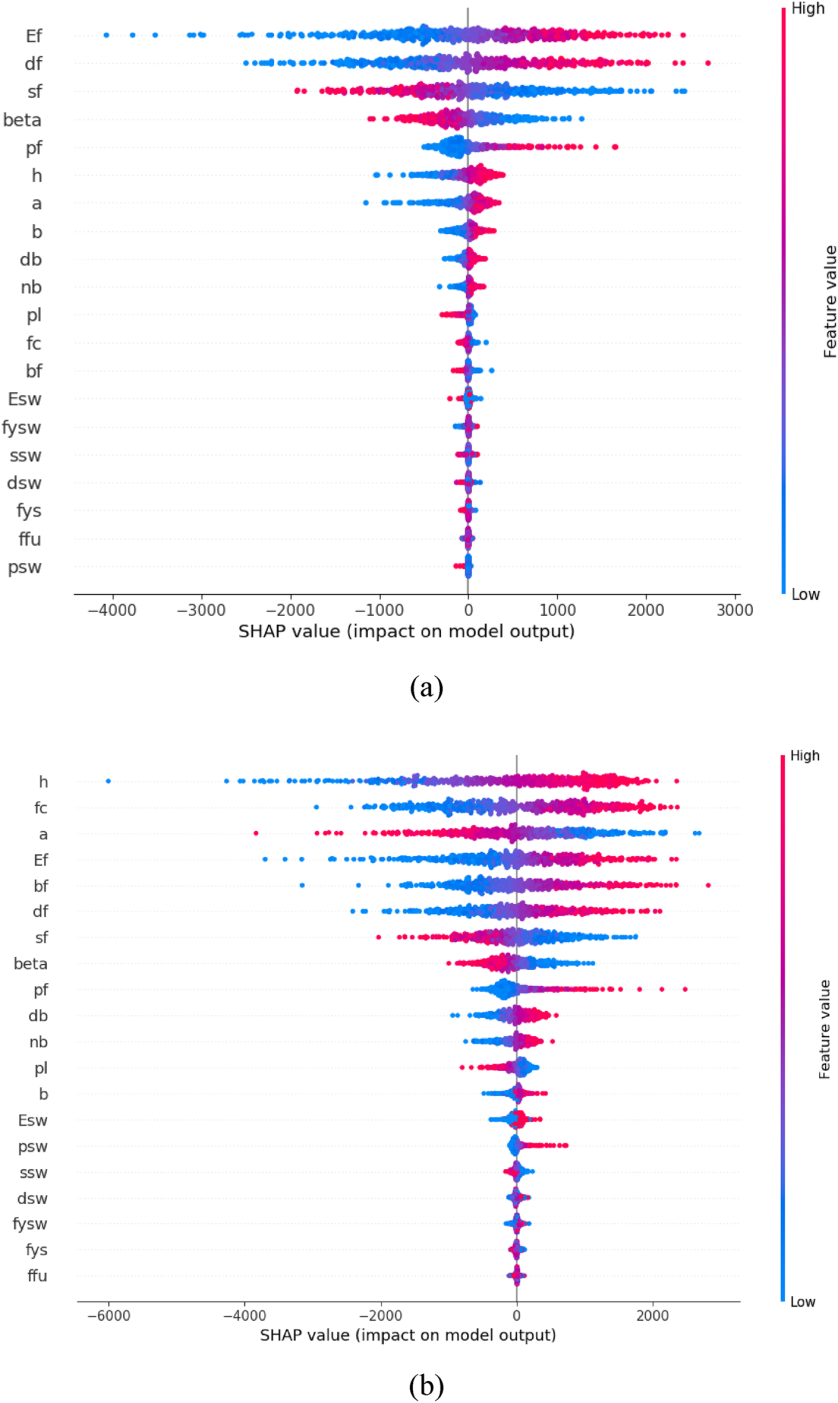
Parametric investigation on (**a**) *V*_*f*_ and (**b**) *V*_*total*_*.*

The robustness and applicability of the proposed methodology were further investigated. Shapley additive explanations were used to assess the influence of design variables on both the shear strength and ETS shear contribution of the beam. The evaluations were conducted using the most proficiently trained ANN models (Fig. 14). The total shear strength of the ETS-strengthened beams (*V*_*total*_) proportionally increased with *b*, *b*_*f*_, *d*_*b*_, *E*_*f*_, *f*_*ys*_, *h*, *f’*_*c*_, *n*_*b*_, and *ρ*_*f*_ but decreased with *a*, *β*, and *s*_*f*_. These trends are well suited to the mechanics domain and existing shear models. For instance, the higher *b*, *b*_*f*_, and *h* are, the larger the beam geometries, resulting in greater shear resistance. Meanwhile, a large *s*_*f*_ enhances the beam action and leads to a small amount of ETS-FRP strengthening, reducing the beam shear strength. Additionally, the shear contribution of ETS strengthening bars (*V*_*f*_) was clearly enhanced with *a*, *b*, *E*_*f*_, *h*, and *ρ*_*f*_ but decreased with *β* and *s*_*f*_. These trends can be explained by the formulations used for *V*_*f*_ in the 2PKT approach, which are suited for the shear resisting mechanism of ETS-strengthened beams. For example, *V*_*f*_ is dependent on the properties and percentages of the ETS strengthening system. On the basis of the parametric investigation, the trained ML models can learn the shear mechanism of ETS-strengthened beams and precisely predict *V*_*f*_ and *V*_*total*_.

## Conclusions

The primary objective of the present study was to propose a new approach for combining the developed 2PKT with ML methods to predict the shear behavior of RC beams strengthened with ETS-FRP bars. The significance of the study involves the contribution of a new computation method to the strengthening field. Consequently, researchers and engineers can utilize the code established for the proposed approach in the design practice of ETS-FRP-strengthened beams without the use of complicated numerical tools. The main conclusions drawn from this work can be summarized as follows:

1. The 2PKT developed in this study can rationally predict the shear behavior of ETS-strengthened RC beams in terms of the beam shear strength, full load–deflection curve, and failure mode. The average *V*_*total-ana.*_/*V*_*total-exp.*_ was 0.98, and the CoV of the mean was 16.2%. The developed 2PKT approach also entails rapid and simple calculation and the possibility of data generation under various design scenarios.
2. On the basis of the analyses of the data derived from the various ML models, random forest failed to predict the shear resistance of ETS-strengthened RC beams. In contrast, XGBoost, LightGBM, and ANN demonstrated great accuracy in predicting the shear contribution of the ETS-FRP strengthening system. The ANN model outperformed the other models in estimating the total shear strength of ETS-strengthened RC beams. In fact, for predicting the total shear strength of ETS-strengthened beams, the ANN model achieved R^2^ values of 0.99, 0.98, and 0.96 for training, validation, and testing data, respectively. These findings suggest that the ANN model is a stable and reliable model for predicting the shear strength of ETS-FRP-retrofitted beams.
3. The studied ML algorithms involve numerous hyperparameters and a wide range of potential values. Bayesian optimization techniques can be used to optimize the hyperparameters of ML models and achieve optimal accuracy. Furthermore, optimizing the ML algorithms promotes an equitable performance comparison of the models.
4. The parametric investigation provides insights into the effects of design variables on the shear capacity of ETS-strengthened RC beams. For example, the total shear strength increased with beam geometry and material strength, while the ETS shear contribution was dependent on the properties and configurations of the FRP. These findings fully agree with those reported in the literature and predicted by the existing shear models.
5. The application of the developed 2PKT–ML approach requires a solid beam and ML theoretical background. Practitioners can apply the developed model by using computational code for predicting the shear behavior of ETS-FRP-strengthened RC beams. A practical tool of the developed 2PKT–ML approach will be established in future studies. The aim is to conveniently serve practitioners in estimating the shear capacity of ETS-FRP-retrofitted beams.
6. The experimental data of the beams strengthened with ETS techniques are still deficient with respect to important design variables, such as ETS material types, ETS configurations, and anchorage systems. Future experimental studies on the ETS strengthening method for RC beams are needed to provide a broad database for evaluating the proposed 2PKT–ML model.

## Data Availability

The datasets generated during and/or analyzed during the current study are available from the corresponding author on reasonable request.
